# Preclinical assessment of an anti-HTLV-1 heterologous DNA/MVA vaccine protocol expressing a multiepitope HBZ protein

**DOI:** 10.1186/s12985-023-02264-z

**Published:** 2023-12-19

**Authors:** D. S. O. Daian e Silva, L. J. Cox, A. S. Rocha, Á. Lopes-Ribeiro, J. P. C. Souza, G. M. Franco, J. L. C. Prado, T. A. Pereira-Santos, M. L. Martins, J. G. A. Coelho-dos-Reis, T. M. Gomes-de-Pinho, F. G. Da Fonseca, E. F. Barbosa-Stancioli

**Affiliations:** 1 Laboratório de Virologia Básica e Aplicada, Departamento de Microbiologia, Instituto de Ciências Biológicas, Universidade Federal de Minas Gerais, Av. Antônio Carlos, 6627, Campus Pampulha, Belo Horizonte, MG CEP 31270-901 Brazil; 2 GIPH – Grupo Interdisciplinar de Pesquisas em HTLV, Interdisciplinary HTLV Research Group, Belo Horizonte, Brazil; 3 Gerência de Desenvolvimento Técnico Científico, Fundação Centro de Hematologia e Hemoterapia do Estado de Minas Gerais – Hemominas, Belo Horizonte, Brazil; 4 Centro de Tecnologia de Vacinas, Universidade Federal de Minas Gerais (UFMG), Belo Horizonte, 31270-901 Brazil

**Keywords:** Vaccine, Human T-lymphotropic virus 1, HBZ, Recombinant Modified *Vaccinia virus* Ankara (MVA-HBZ), Plasmid DNA vaccine (pcDNA3.1(+)-HBZ)

## Abstract

**Background:**

*Human T-lymphotropic virus 1* (HTLV-1) is associated with the development of several pathologies and chronic infection in humans. The inefficiency of the available treatments and the challenge in developing a protective vaccine highlight the need to produce effective immunotherapeutic tools. The HTLV-1 basic leucine zipper (bZIP) factor (HBZ) plays an important role in the HTLV-1 persistence, conferring a survival advantage to infected cells by reducing the HTLV-1 proteins expression, allowing infected cells to evade immune surveillance, and enhancing cell proliferation leading to increased proviral load.

**Methods:**

We have generated a recombinant Modified *Virus Vaccinia* Ankara (MVA-HBZ) and a plasmid DNA (pcDNA3.1(+)-HBZ) expressing a multiepitope protein based on peptides of HBZ to study the immunogenic potential of this viral-derived protein in BALB/c mice model. Mice were immunized in a prime-boost heterologous protocol and their splenocytes (T CD4^+^ and T CD8^+^) were immunophenotyped by flow cytometry and the humoral response was evaluated by ELISA using HBZ protein produced in prokaryotic vector as antigen.

**Results:**

T CD4^+^ and T CD8^+^ lymphocytes cells stimulated by HBZ-peptides (HBZ_42–50_ and HBZ_157–176_) showed polyfunctional double positive responses for TNF-α/IFN-γ, and TNF-α/IL-2. Moreover, T CD8^+^ cells presented a tendency in the activation of effector memory cells producing granzyme B (CD44^+High^/CD62L^−Low^), and the activation of Cytotoxic T Lymphocytes (CTLs) and cytotoxic responses in immunized mice were inferred through the production of granzyme B by effector memory T cells and the expression of CD107a by CD8^+^ T cells. The overall data is consistent with a directive and effector recall response, which may be able to operate actively in the elimination of HTLV-1-infected cells and, consequently, in the reduction of the proviral load. Sera from immunized mice, differently from those of control animals, showed IgG-anti-HBZ production by ELISA.

**Conclusions:**

Our results highlight the potential of the HBZ multiepitope protein expressed from plasmid DNA and a poxviral vector as candidates for therapeutic vaccine.

**Supplementary Information:**

The online version contains supplementary material available at 10.1186/s12985-023-02264-z.

## Background

HTLV-1 (*Human T-lymphotropic virus 1*) is a retrovirus that infects about 20 million people worldwide with epidemiological data pointing to Brazil as the country with the largest absolute number of infected individuals, around to 2 million infected people [1]. However, it is estimated that the real prevalence of HTLV-1 in Brazil and around the world is higher than the data found in the literature. Most of individuals after infection remain as asymptomatic carriers (~ 90 to 95%) and are potential disseminators, while less than 10% develop diseases with unfavorable outcomes, among them HTLV-Associated Myelopathy/Tropical Spastic Paraparesis (HAM/TSP), a chronic inflammatory neurodegenerative disease with important morbidity for the patients [2, 3], and about 1–5% of them undergo clonal expansion of infected CD4+ T-cells and develop ATL after a long latency period. Detection of oligoclonal and monoclonal expansion of infected cells via analysis of proviral integration sites and/or profiling of T cells receptor repertoire is documented as a risk factor for ATL onset and development [4–6].

In addition to these, other inflammatory diseases can be associated with HTLV-1 infection, such as uveitis, infectious dermatitis, polymyositis, arthritis, alveolitis and Sjögren's syndrome [2]. A meta-analysis study based on epidemiological data on associations between health outcomes and HTLV-1 showed an increased relative risk of premature death in individuals infected with HTLV-1 independent of ATL and HAM/TSP occurrence, and it was related to the illness’s prevalence in HTLV-1 infected people, that contribute to the poorer survival of them [7]. A Spanish study observed that common age-related diseases, such as cardiovascular events, neurodegenerative diseases, metabolic abnormalities, and osteoporosis have occurred consistently in HTLV-1 carriers. The authors proposed that chronic inflammation and accelerating aging might have occurred earlier in HTLV-1 infected people compared to noninfected individuals and that these conditions are responsible for shortened survival in HTLV-1 infected individuals, even in those who do not develop ATL or HAM/TSP [8]. Preventive and therapeutic approaches against HTLV-1 infection and related pathologies are limited. Therefore, the development of a novel anti-HTLV-1 vaccine is still required.

Based on the knowledge advances in the HTLV-host interaction and in the biotechnology recent systematic reviews and reports showed a plethora of approaches for HTLV-1 vaccine candidates: viral like particles vectors and viral vectors (Vaccinia virus, Adenovirus and Sendai virus), dendritic cell and peptide-based vaccines, recombinant single proteins or multiepitope proteins expressed in different systems including molecules encapsulated using nanotechnology [9–13]. Of great interest 15 peptide stretches (from Gag and Gag-Pro-Pol polyproteins, Tax-1 and gp62) were presented from an epitope platform for HTLV-1 vaccine development of uniquely viral non-human pentapeptides to avoid cross-reaction, generating safe immunization based on peptides and mRNA [14].

The poxvirus vectors are considered safe to be used as recombinant vaccines. These vectors immunogenicity comparable to a live virus vaccine, by expressing gene products that are efficiently presented by both MHC-I and MHC-II molecules, therefore leading to the activation of both CD8 and CD4 antigen-specific and the innate immune responses, that is crucial to mediate the reduction of the risk of viral acquisition [15]. Previous studies conducted with the use of ALVAC-Env administration reduced the risk of HTLV-1 infection in the rabbit model [16]. Sugata et al. [17] proved the increase survival of the ATL mice model after vaccination with recombinant *Vaccinia virus* expressing HBZ (rVV-HBZ) that induced a cytotoxic response against cells that express HBZ in vivo. According to recent review, MVA is one of the most used, safe and a very promising vaccine vector against cancer and different infectious diseases, including AIDS, tuberculosis, and Ebola. Authors provide information about clinical trials already in progress using this vector and its new generation, with improvement of the immunogenicity and efficacy [18].

One of the protagonists in the HTLV-1 chronic infection scenario is the HTLV-1 basic leucine zipper (bzip) factor (HBZ), a regulatory HTLV-1 protein encoded by the antisense strand in the proviral DNA. HBZ is associated with cell proliferation and survival, inflammatory responses, immune evasion, apoptosis and autophagy repression, and genomic instability, and therefore it could be a promising therapeutic target [17, 19–22]. The present study evaluated the immunogenic potential of generated recombinant MVA and DNA plasmid expressing an HBZ multiepitope protein as a candidate for therapeutic vaccine against HTLV-1.

## Material and methods

### Design of the recombinant virus MVA-HBZ

The multiepitope protein design was based on HBZ sequence deposited in the GenBank database [17, 23, 24], and in silico analyses performed using the epitope prediction tools: NetMHCpan 4.0 (Threshold: weak binders—2.0; strong binders—0.5), NetCTL 1.2 (Threshold of 0.75) and IEDB—MHC-I Binding Predictions.

Epitopes linked to the HLA-A*02 allele (considered protective in HTLV-1 context) were chosen based on the scores obtained from the three tools. Alignment of all deposited sequences was performed using the BioEdit Sequence Alignment Editor 7.2.5 to verify the conservation of epitopes.

**Fig. 1:**
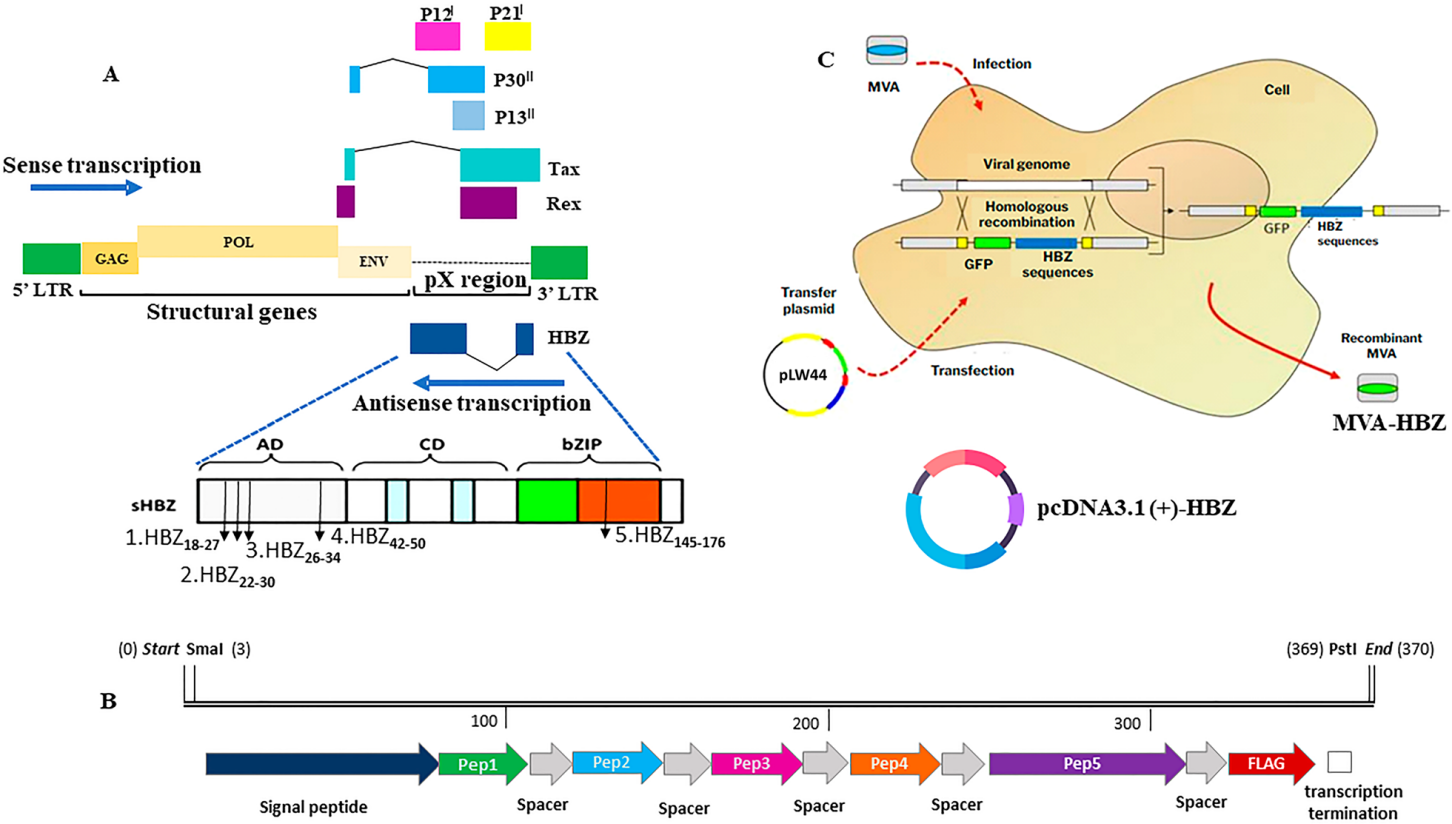
Schematic diagram of HTLV-1 proviral genome and the HBZ multiepitope cassette construction. **A** HTLV-1 proviral genome pointing out the HTLV-1 basic leucine zipper factor (HBZ) in the antisense strand. The sHBZ box demonstrate in the Activator Domain (AD) and bZIP region the position of peptides 1 to 3 (in silico analysis), peptide 4 (MacNamara et al. [23]), and peptide 5 (Sugata et al. [17]) that compound the multiepitope recombinant protein. **B** HBZ multiepitope cassette presenting the selected peptides (pep1 to pep5), spacer regions (GSGSG), signal peptide (MDAMKRGLCCVLLLCGAVFVDSVTG), and a flag tag (DYKDDDDK). **C** Construction of recombinant MVA-HBZ by homologous recombination expressing the HBZ multiepitope protein. A pLW44 plasmid that contains the gene of interest is used to transfect an MVA-infected cell. The pcDNA3.1(+)-HBZ express the same HBZ multiepitope protein

Following the design of the HBZ-multiepitope sequence, a signal peptide was added [25] as well as flexible spacers composed of glycine and serine “GSGSG” [26] in between peptides. The construction was designed using the SnapGene 1.4.1 program. Prediction of the signal peptide was performed using the SignalP 4.1 tool. Additionally, it was added a FLAG tag sequence “DYKDDDDK” for in vitro assays, a transcription terminator for MVA “TTTTTGT”, and a translation terminator “TAA” (Fig. 1). The sequence was synthesized in the vector pCloneEZ-NRS-Blunt-Amp by GenOne (Brazil) and subcloned in pLW44 transfer plasmid. The recombinant virus sequence includes four epitopes from central domain (CD) and one epitope from basic leucine zipper (bZIP) domain regions of HBZ and it is under intellectual protection (BR1020190183934), reason why the sequence will not be described here.

### Recombinant virus production

The recombinant viral vector was selected, amplified, and titrated in baby hamster kidney cells (BHK-21—Baby Hamster Kidney fibroblasts, originally derived from the American Type Culture Collection—ATCC® CCL-10™). The synthetic gene was inserted into a transfer plasmid (containing the green fluorescent protein sequence for viral in vitro selection) under the control of the mH5 early late *Vaccinia virus* promoter [27]. The construction of the recombinant vaccine vector was based on the homologous recombination between the transfer plasmid, containing the nucleotide sequences coding for the HBZ multiepitope protein and the parental MVA (wt—wild type) DNA sequences. Recombinant clones were selected using the aid of the GFP marker and the construction was validated through DNA sequencing, detection of transcripts (by RT-PCR using oligoDT and HBZ specific primers) after infection of BHK-21 cells with MVA-HBZ (multiplicity of infection [MOI] of 1), and protein detection through Western Blot (data not shown).

For viral stock production, cells were infected at 37 °C, 5% CO_2_ atmosphere for 1 h. After adsorption, the cells were incubated in the same atmosphere in DMEM (Dulbecco's Modified Eagle Medium—Sigma Aldrich, USA) supplemented with 7.5% NaHCO_3_, antibiotics (100 μg/mL streptomycin and 100 U/mL penicillin), antifungal (fungizone at 25 μg/mL) and 5% fetal bovine serum (FBS—Gibco, USA) for 48 h. Recombinant virus purification was performed in 36% (w/v) sucrose cushion (in 10 mM Tris–HCl, pH 9.0) and the titer was determined by a plaque-based assay [28, 29].

### Recombinant pcDNA3.1(+)-HBZ plasmid production

A pcDNA3.1(+) encoding the HBZ multiepitope protein gene and a signal peptide, the same as the recombinant virus, was constructed by FastBio (Brazil) (Additional file 1: Fig. S1). The plasmid was cloned, amplified, and purified in *Escherichia coli* DH5α cells with 100 μg/mL ampicillin and incubated for 16–18 h at 37 °C. Colonies were isolated and tested for the presence of the segment of interest by PCR (T7 *Promoter* [*Forward*] and M13 [*Reverse*]) and enzymatic restriction (*NheI* and *SmaI*). The amplified and restricted products were fractionated in 1% agarose gel in TBE (tris–borate-EDTA), treated with SybrSafe (Thermo Scientific), and visualized on the ChimiDocTM Gel Imaging System (Bio-rad).

Alternatively, the plasmid was purified using the QIAprep Spin Miniprep Kit (Qiagen, USA), according to the manufacturer's instructions. A specific purification process was carried out aiming at the removal of bacterial endotoxins using the EndoFree Plasmid Giga Kit (Qiagen), according to the manufacturer's instructions and.

### HBZ recombinant protein

A pET21a(+) presenting the HBZ complete protein was synthetically constructed by GenOne (Brazil). The plasmid was cloned, amplified, and purified in *Escherichia coli* DH5α cells with 100 μg/mL ampicillin and incubated for 16–18 h at 37 °C. Colonies were isolated and tested for the presence of the segment of interest by PCR (T7 Promoter [*Forward*] and T7 Terminator [*Reverse*]). The amplified products were fractionated in 1% agarose gel in TBE (tris–borate-EDTA), treated with SybrSafe (Thermo Scientific) and visualized on the ChimiDocTM Gel Imaging System (Bio-rad). Alternatively, the plasmid was purified using the QIAprep Spin Miniprep Kit (Qiagen, USA) according to the manufacturer's instructions. The expression of HBZ was induced in *Rosetta-gami* 2(DE3) and the protein was purified in the Äkta Start system (GE Healthcare Life Sciences, Sweden) with imidazole 20 mM and urea 8 M binding buffer. Elution was performed with imidazole 500 mM. Protein expression was evaluated through 12% SDS-PAGE and Western Blot (data not shown).

### In vitro expression of HBZ: analysis by flow cytometry

Flow cytometry assays were performed to detect the in vitro expression of HBZ multiepitope protein by the recombinant MVA virus and the plasmid. For this, BHK-21 cells were infected with MVA-HBZ in a MOI of 1, or transfected (5 µg) with the pcDNA3.1(+)-HBZ recombinant plasmid for 24–48 h. After the incubation period, the cells were counted and plated on a 96-well cell culture plate with “U” bottom (Falcon® 96-Well Round-Bottom Plates, Corning®) and adjusted to 4 × 10^6^ cells/mL in a DMEM medium supplemented with 2.5% FBS and antibiotics, as described above. Cells were stained with anti-Flag antibody (*Sigma-Aldrich*—F3165) and with anti-mouse IgG TRITC (*Sigma-Aldrich*) at 37 °C for 30 min each. Fixation was performed with 1% paraformaldehyde.

The samples were acquired in a flow cytometer FACSCantoTM II (BD Biosciences), being acquired up to 100,000 total events by the BD FACSDiva 6.1 acquisition software. Data analysis was performed using the FlowJo program (BD Biosciences). Non-specific results were assessed using a control virus (MVA-GFP) and unstained samples. The graphics were produced using the GraphPad Prism 8.0 software (GraphPad Inc., San Diego, CA, USA).

### Mice immunization with recombinant virus or plasmid

BALB/c female mice, 8 weeks old, obtained from the animal facility at the Universidade Federal de Minas Gerais (UFMG), were adapted in mini-isolators housed in ventilated racks for seven (07) days in the maintenance facility, being submitted to a light/dark cycle, as well as, with free access to food and water. Immunizations were performed in animals previously anesthetized intraperitoneally (i.p.) with ketamine and xylazine (75 mg/kg and 10 mg/kg, respectively).

**Fig. 2:**
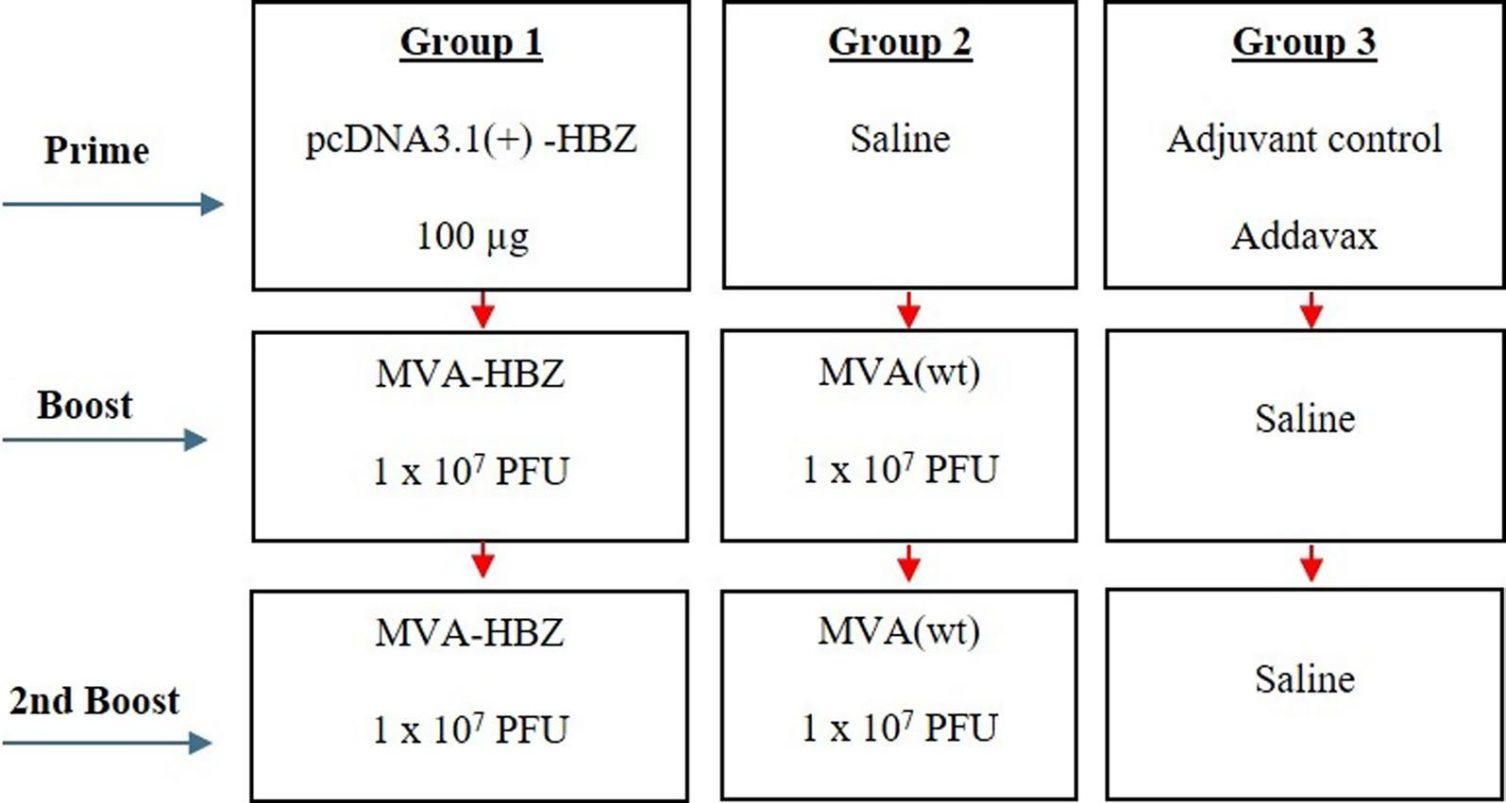
Mice immunization protocol. Group 1 received: Prime—pcDNA3.1(+)-HBZ (100 µg); Boost—MVA-HBZ 10^7^ PFU and 2nd Boost—MVA-HBZ 10^7^ PFU. Group 2 received: Prime—Saline; Boost—MVA (wt) 10^7^ PFU and 2nd Boost—MVA (wt) 10^7^ PFU. Group 3 received: Prime—Addavax 1:1; Boost—Saline and 2nd Boost—Saline. N = 7. Immunizations were performed at 14 days-intervals

The animals from group 1 were immunized by intramuscular injection (quadriceps muscle), using an insulin needle (31G), with 100 µg of recombinant plasmid with Addavax adjuvant 1:1 and 10^7^ plaque-forming units (pfu) of MVA-HBZ. Fourteen days after, they received another injection (second boost) with 10^7^ pfu of MVA-HBZ (pcDNA3.1(+)-HBZ_MVA_HBZ__MVA_HBZ_). As a control, other two groups of mice were immunized with MVA (wt) (group 2: saline_MVA[wt]_MVA[wt]) or with saline (group 3: Addavax_saline_saline) as prime and second boost (Fig. 2). After 29 days, the animals were euthanized and blood and spleens were collected for further analysis, as described below [30].

### Immunophenotyping and intracellular cytokine staining (ICS)

The immunophenotyping and ICS protocols used in this study were adapted from Quinan et al. [31]. Briefly, cells from mice euthanized after 29 days were used for cellular analysis. Cultures of splenocytes (1 × 10^7^ cells) were stimulated with a pool of HBZ peptides (1,5 µg/mL for every 10^6^ cells) at 37 °C with 5% CO_2_. The pool of HBZ peptides (HBZ_42–50_ and HBZ_157–176_) was based on the previous studies [17, 23]. The cells were also treated with Fc Block (Purified Rat Anti-Mouse CD16/CD32—BD) to reduce non-specific bindings. For positive controls we used *Vaccinia virus* peptide A3 and PMA (phorbol 12-myristate 13-acetate)/Ionomycin eBioscience™ Cell Stimulation Cocktail (Thermo Scientific). For background control, it was used wells with no stimulation.

Cells were stimulated for 4 h with stimuli, 10 μg/mL of Brefeldin A (eBioscience™ Brefeldin A Solution [1000×]—Thermo Scientific) and CD107a-FITC (clone 1D4B, *BD Biosciences*). EDTA to a final concentration of 2 mM per well was added 15 min before the end of the incubation. After washing, cell surface staining was performed using: *Live/Dead®* violet fluorescent reactive dye (Invitrogen) for viability, CD8α-PerCP (clone 53–6.7, *BD Biosciences*), CD4-PE (clone GK1.5, *BD Biosciences)*, CD3-Brilliant Violet 711 (clone 145-2C11, *Biolegend*), CD62L-APC (clone MEL-14, *Biolegend*) and CD44-FITC (clone IM-7, *Biolegend*).

The intracellular cytokine labeling was performed using monoclonal antibodies for the specific targets-fluorochromes: IFN-γ-APC (clone XMG1.2, *Biolegend*)*,* IL2-PE_Cy7_ (clone JES6-5H4, *Biolegend)*, TNF-α-APC_Cy7_ (clone MP6-XT22, *Biolegend*) and granzyme B-PE (clone NGZB, Invitrogen). Fixation was performed with 1% paraformaldehyde solution. Results were acquired using LSRFortessa™ (BD Biosciences), with up to 100,000 events being acquired using BD FACSDiva 6.1 acquisition software.

The data were analyzed with FlowJo software (BD Biosciences) and the ratio between the background values ​​and that ​​presented by the tested samples was performed. The graphs were produced using GraphPad Prism 8.0 software (GraphPad Inc., San Diego, CA, USA) and data are represented as mean and standard error of the mean (SEM).

### Indirect ELISA for analysis of humoral response in immunized mice

Indirect in-house enzyme-linked immunosorbent assay (ELISA) was used for the evaluation of the humoral immune response in sera from immunized mice. For this, 96 well plates (Greiner High Binding) were coated at 4 °C overnight with 150 ng/well of HBZ recombinant protein. Blocking was made with 5% BSA (bovine serum albumin—Fitzgerald—30-AB70) for 16–18 h at 4 °C. Next, mice sera were diluted 1:50 in washing solution (BSA 0.1%) and the plates were incubated at 37 °C for 1 h (h) in humid chamber. Next, the plates were washed and incubated with anti-mouse IgG conjugated to peroxidase (anti-mouse IgG peroxidase whole molecule [WM]—A9044, Sigma Aldrich, EUA), diluted 1:10.000 and incubated for 1.5 h at room temperature in humid chamber. Revelation was performed using TMB as a substrate (Tetramethylbenzidine. One Step-TMB Linear [Scienco]) and 2 M H_2_SO_4_ as stop solution. The plates were read at 450 nm absorbance in a spectrophotometer (Thermo Scientific™ Multiskan™ GO Microplate Spectrophotometer).

### Statistical analysis

The results were analyzed using the GraphPad Prism 8.0 program (GraphPad Inc., San Diego, CA, USA) and the data are represented as the mean and standard error of the mean (SEM). Statistical analyses were performed using Kruskal–Wallis for multiple comparisons or Mann–Whitney test for comparisons between two groups (GraphPad Inc., San Diego, CA, USA). Differences were considered significant when *p* < 0.05.

## Results

### Construction of the recombinant vectors

In this study, two vaccine prototypes based on a plasmid vector (pcDNA3.1[+]-HBZ) and a poxvirus recombinant vector (MVA-HBZ) were designed with the purpose of developing a heterologous protocol that could be used as a therapeutic option in the treatment of HTLV-1-infected patients.

**Fig. 3:**
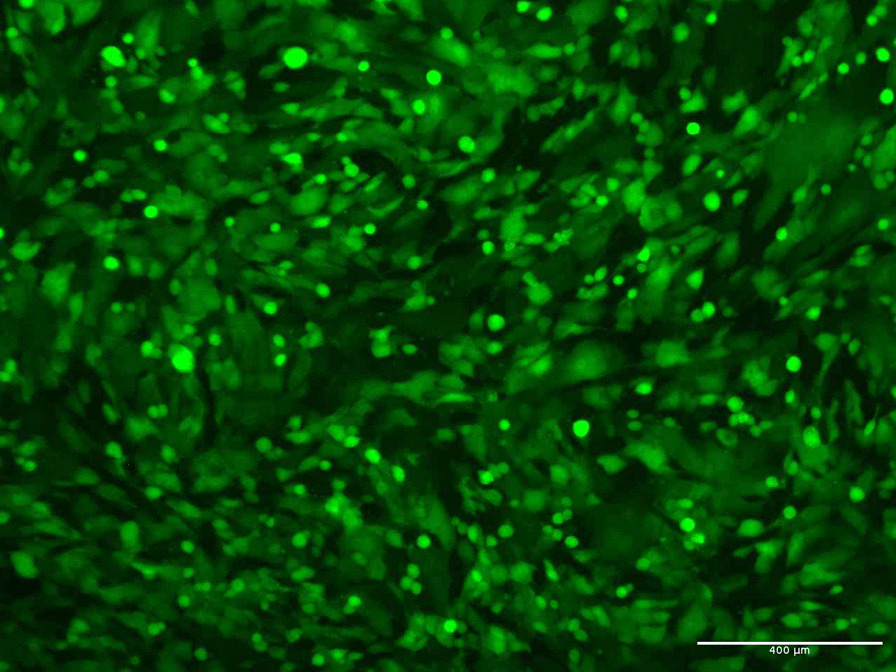
Microscopy of MVA-HBZ infected foci in BHK-21 cells monolayer. Fluorescence microscopy shows green fluorescence, due to GFP-tag 24 h post-infection in BHK-21 cells. Fluorescence microscope EVOS® FL Cell Imaging System (Thermo Fisher), at 400× magnification

The recombinant virus MVA-HBZ was developed through a process of homologous recombination between a transfer plasmid and MVA (wt) in eukaryotic cells. Recombinant viral clones were identified with the aid of GFP expression (fluorescent foci) and evaluated by PCR, as well as through nucleotide sequencing (data not shown). Through this set of analyses, it was possible to select the viral clones that presented the desired recombinant sequence. An illustrative image of the MVA viral vector in BHK-21 cell culture is shown in Fig. 3.

The multiepitope plasmid pcDNA3.1(+)-HBZ was cloned into *Escherichia coli* DH5α cells for amplification and purification. Transforming colonies were selected by the identification of the complete recombinant plasmid sequence using PCR and the multiepitope sequence using restriction enzymes (supplementary Figure S2). Both assays showed the expected fragments of 2702 bp and 1437 bp, respectively. Next, the plasmids were endotoxin purified, and the integrity of the insert was evaluated by PCR, showing an expected fragment of 2702 bp (supplementary Figure S3). All vectors were positive for the presence of the multiepitope DNA sequence and were used in the next steps.

### MVA-HBZ and pcDNA3.1(+)-HBZ efficiently express HBZ multiepitope protein

**Fig. 4:**
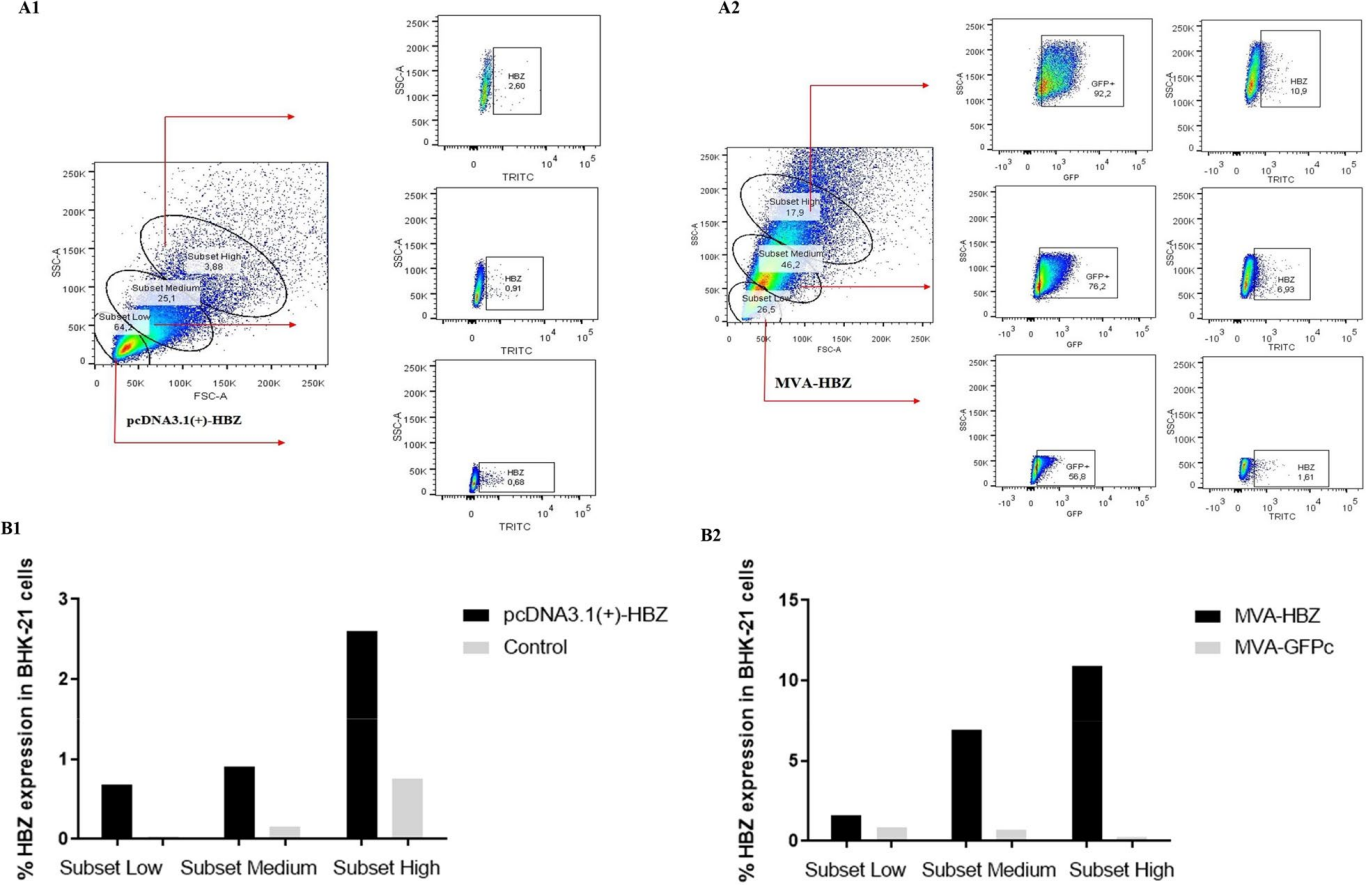
Analysis of HBZ multiepitope protein expression by the pcDNA and MVA vector. **A** Representative graphs of the gating strategy used in the flow cytometry expression assays. Total percentage of HBZ expression in BHK-21 transfected / infected cells. Subset low (subset low), subset (subset medium) and subset high (subset high) according to cell size (FSC) and granularity (SSC). **A1** for pcDNA3.1(+)-HBZ and **A2** for MVA-HBZ. **B**–**B1** In black: pcDNA3.1(+)-HBZ; In gray: Mock transfected cell control. **B2** In black: MVA-HBZ; In gray: MVA-GFP control. Both vectors showed efficient recombinant protein expression in vitro mainly in subsets medium and high

The analysis of protein expression by the recombinant vectors was performed through infection and transfection assays in BHK-21 cells and labeling using a FLAG tag present in the recombinant sequence (Fig. 4). It was possible to certify that both, recombinant virus and plasmid, efficiently express the recombinant multiepitope HBZ protein in vitro. MVA-GFP virus was used as a background control, as well as non-stained samples.

For the gating strategy, the cell subsets were divided into low, medium and high subsets, according to cell sizes (FSC—forward scatter) and granularity (SSC—side scatter).

The analysis contemplates different cell subsets that are in distinct stages of the cell cycle (different sizes and granularity) which, in turn, also express the evaluated antigen in a varied way. For pcDNA3.1(+)-HBZ, all three subsets showed high expression of the analyzed antigen, with the medium and high subsets standing out probably due to advances in the cell maturity state and highly active metabolism. The same was observed for cells infected with the MVA-recombinant virus. It is noteworthy that both transfection and viral infection interfere with internal mechanisms and cell morphology, thus likewise reflecting on the different expression patterns observed for the cell subsets evaluated.

### Heterologous vaccination protocol with HBZ multiepitope protein induces cytokine-producing CD8^+^ T cell and cytotoxic response in BALB/c mice

The preclinical evaluation of the HBZ multiepitope protein, conveyed by two different vectors, to induce immune response was evaluated through the immunization of BALB/c mice (n = 7) in a prime-boost heterologous protocol (Fig. 2). Twenty-nine days after the last booster dose, mice were euthanized and the spleens were collected for multiparametric flow cytometry analysis under stimulation with HBZ-derived peptides.

**Fig. 5:**
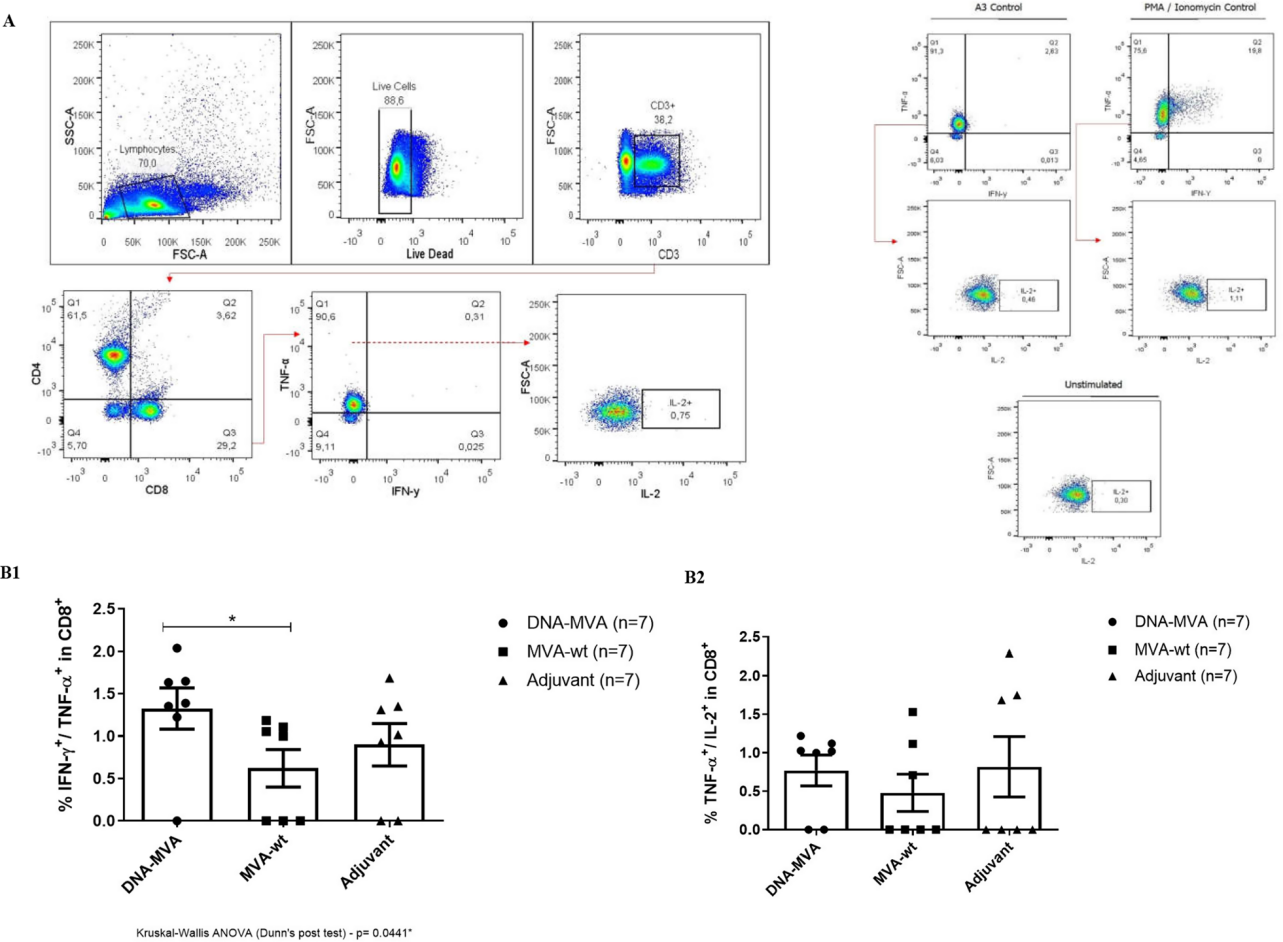
Recruitment of T CD8^+^ lymphocytes and cytokine production in mice immunized with the recombinant DNA-MVA. **A** Representative graphs of the gating strategy used in the flow cytometry assays. The A3 peptide and PMA / Ionomycin were used as positive controls. **B**–**B1** Graph of the total percentage of CD8^+^ T lymphocytes, double-positives for INF-γ and TNF-α recruited after restimulation. Statistical differences were observed between the groups DNA-MVA vs MVA (wt). **B2** Graph of the total percentage of T CD8^+^ lymphocytes producers of TNF-α and IL-2 recruited after restimulation with HBZ peptides. Legend (symbols): Circle: Cells from mice immunized with the recombinant DNA-MVA regimen; Square: Cells from mice inoculated only with the MVA (wt) vector; Triangle: Cells from mice inoculated only with the Addavax adjuvant. N = 7

The quality of the immune response triggered by vaccination was evaluated by the capacity of T CD8^+^ cells to produce three key mediators in the inflammatory process: IFN-γ, TNF-α, and IL-2. As stimulation controls, we used *Vaccinia virus* A3 peptide and PMA/ionomycin. The results are shown in Fig. 5.

Regarding the response of CD8^+^ T cells to vaccination, the results indicate that the proposed prime-booster protocol (DNA_HBZ__MVA_HBZ__MVA_HBZ_) was efficient in recruiting polyfunctional responses, inducing IFN-γ and TNF-α-producing cells, as well as, the secretion of IL-2.

Statistically significant differences were found between the groups DNA-MVA and MVA (wt) for the double positive cells for IFN-γ and TNF-α (Kruskal–Wallis, Dunn's post-test, *p* = 0.0441). However, no differences were observed between the group’s DNA-MVA and adjuvant control group for the double positive cells for IFN-γ and TNF-α (Kruskal–Wallis, Dunn's post-test, *p* = 0.3658). It is also possible to observe an increased tendency of the vaccinated group compared to the adjuvant control group to induce this same type of response.

For IL-2, TNF-α-producing CD8^+^ T cells in the vaccinated group tended to have a higher expression of this cytokine compared to the MVA (wt) control group, although no statistically significant differences were observed between the groups (Kruskal–Wallis *p* = 0.7005). Control mice inoculated only with the adjuvant showed, in general, high convergence to TNF-α expression, indicating a possible inflammatory background that may have been exacerbated by the adjuvant administration.

**Fig. 6:**
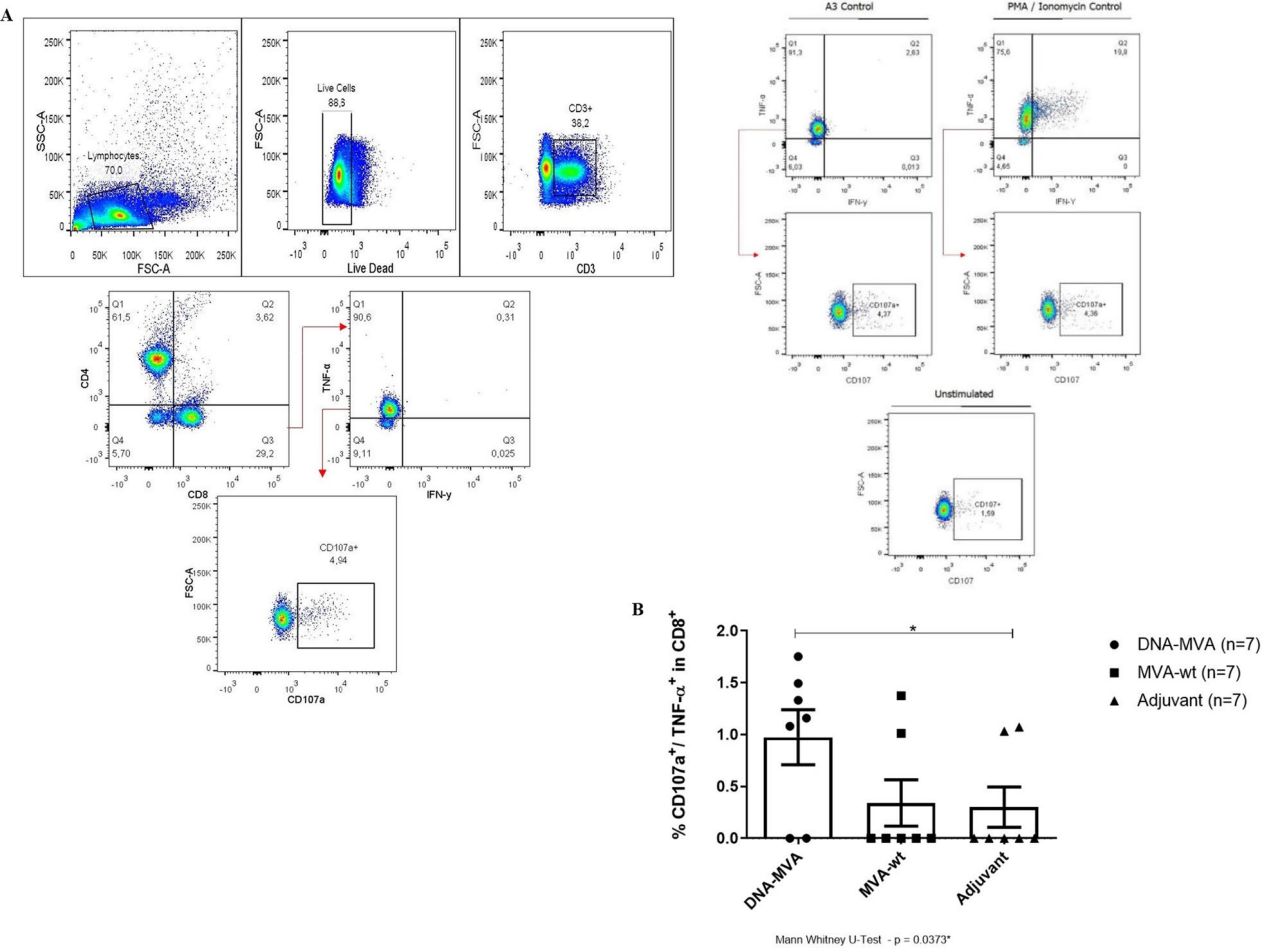
Recruitment of T CD8^+^ lymphocytes, TNF-α and CD107a production in mice immunized with the recombinant DNA-MVA. **A** Representative graphs of the gating strategy used in the flow cytometry assays. The A3 peptide and PMA/Ionomycin were used as positive controls. **B** Graph of the total percentage of T CD8^+^ lymphocytes producers of TNF-α, expressing CD107a, recruited after restimulation with HBZ peptides. Statistical differences were observed between the group’s DNA-MVA vs Adjuvant. Legend (symbols): Circle: Cells from mice immunized with the recombinant DNA-MVA regimen; Square: Cells from mice inoculated only with the MVA (wt) vector; Triangle: Cells from mice inoculated only with the Addavax adjuvant. N = 7

In addition, the cytotoxic CD8^+^ T response was analyzed through the detection of CD107a and granzyme B molecules, after restimulation of cultured cells from spleens of immunized mice with a pool of developed HBZ peptides. When evaluating TNF-α-producing CD8^+^ T cells expressing the activation marker CD107a, statistically significant differences were found between the vaccinated group and the adjuvant control group (Mann–Whitney U test, *p* = 0.0373). The vaccinated group also showed a tendency for cell activation and recruitment compared to the control group MVA (wt) (Fig. 6).

**Fig. 7:**
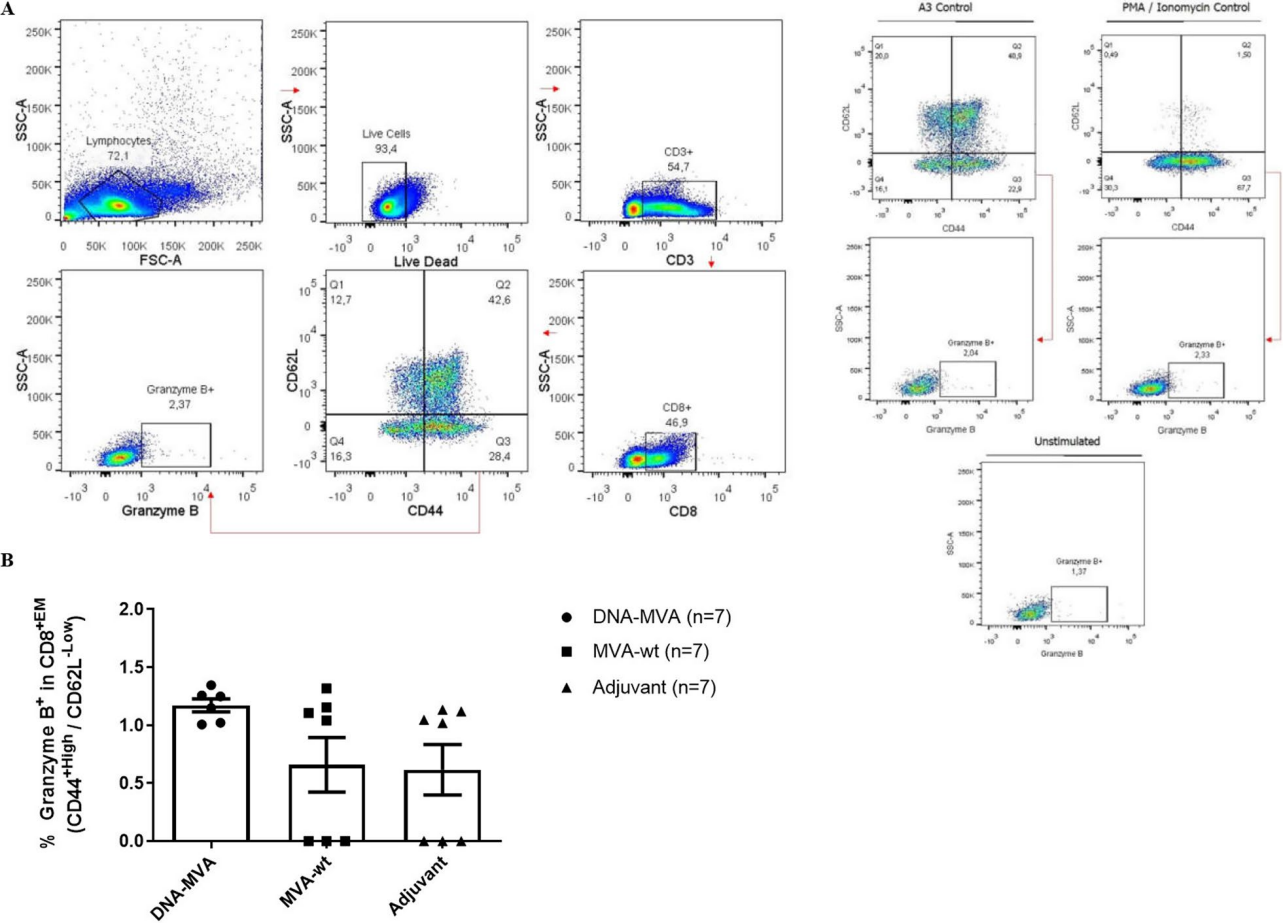
Recruitment of T CD8^+EM^, CD44^+High^/CD62L^−Low^, granzyme B^+^ in mice immunized with the recombinant DNA-MVA. **A** Representative graphs of the gating strategy used in the flow cytometry assays. The A3 peptide and PMA/Ionomycin were used as positive controls. **B** Graph of the total percentage of T CD8^+EM^ lymphocytes CD44^+High^ / CD62L^−Low^ producers of granzyme B recruited after restimulation with HBZ peptides. Legend (symbols): Circle: Cells from mice immunized with the recombinant DNA-MVA regimen; Square: Cells from mice inoculated only with the MVA (wt) vector; Triangle: Cells from mice inoculated only with the Addavax adjuvant. N = 7

A population of interest related to the cytotoxic CD8^+^ T cells consists of the effector memory subgroup, composed of differentiated and dynamic cells with a high capacity for rapid response to their specific antigen. For this population no statistically significant differences were observed between the groups (Kruskal–Wallis *p* = 0.1385). However, Effector memory CD8^+^ T cells (CD44^+High^/CD62L^−Low^) related to the vaccinated group showed a tendency to produce granzyme B compared to the control groups, which is concordant with those data obtained for the CD107a marker since both are associated with the lysosomal granules of cytotoxic response (Fig. 7).

### Heterologous vaccination protocol with HBZ multiepitope protein induces cytokine-producing CD4^+^ T cell and humoral response in BALB/c mice

Post-vaccination immune modulation related to CD4^+^ T lymphocytes is correlated with a plural profile of cytokine production such as IFN-γ, TNF-α and IL-2. T CD4^+^ responses in immunized mice were evaluated by the capacity of these cells to produce IFN-γ, TNF-α, and IL-2 under stimulation of the HBZ peptides. As stimulation controls, we have used *Vaccinia virus* A3 peptide and PMA/ionomycin.

**Fig. 8:**
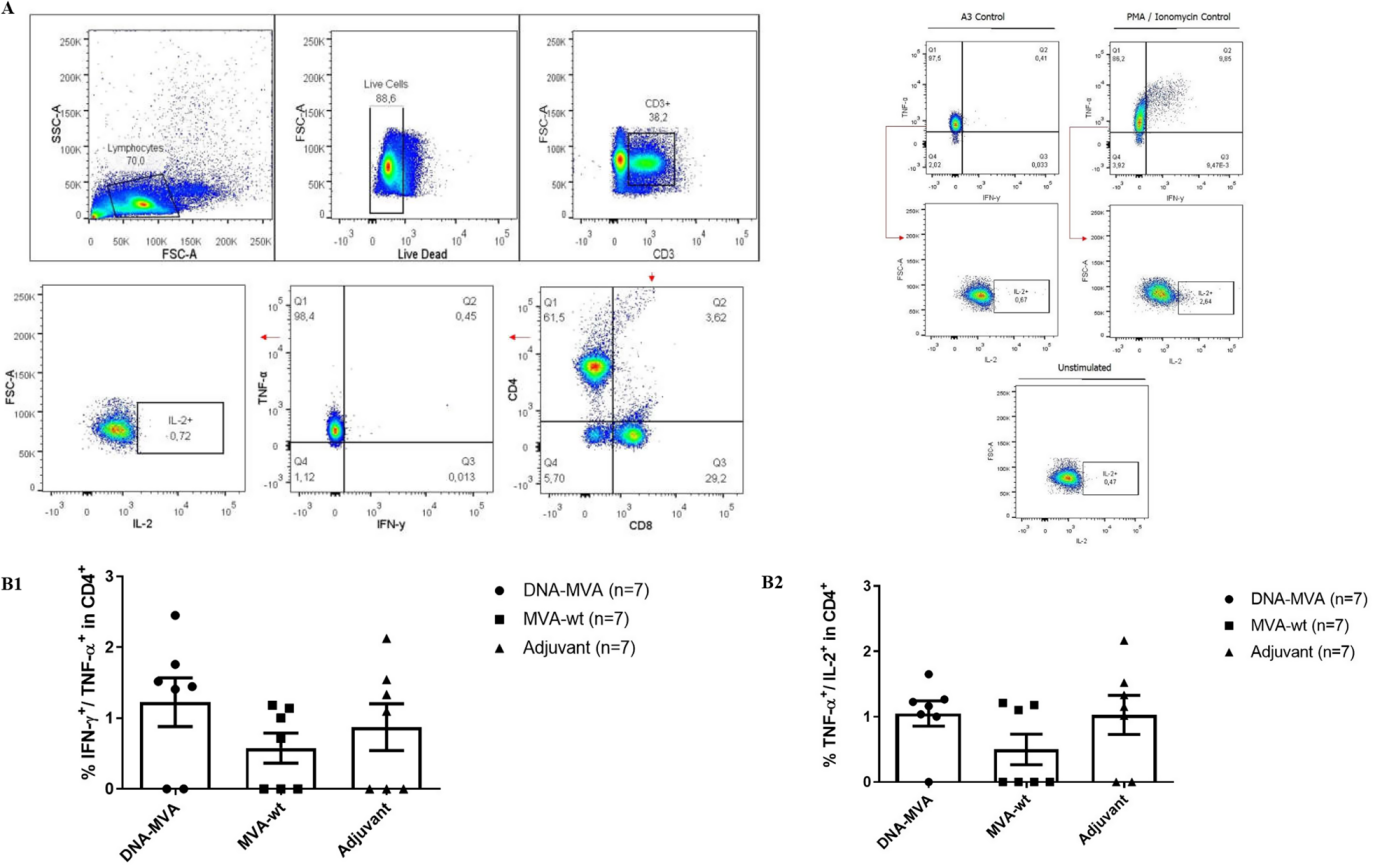
Recruitment of T CD4^+^ lymphocytes and cytokine production in mice immunized with the recombinant DNA-MVA. **A** Representative graphs of the gating strategy used in the flow cytometry assays. The A3 peptide and PMA/Ionomycin were used as positive controls. **B–B1** Graph of the total percentage of CD4^+^ T lymphocytes, double-positives for INF-γ and TNF-α recruited after restimulation. **B2** Graph of the total percentage of T CD4^+^ lymphocytes producers of TNF-α and IL-2 recruited after restimulation with HBZ peptides. Legend (symbols): Circle: Cells from mice immunized with the recombinant DNA-MVA regimen; Square: Cells from mice inoculated only with the MVA (wt) vector; Triangle: Cells from mice inoculated only with the Addavax adjuvant. N = 7

The results indicated that the proposed DNA_HBZ__MVA_HBZ__MVA_HBZ_ vaccine regimen was also efficient in inducing polyfunctional responses, inducing the recruitment of IFN-γ, TNF-α and IL-2 produced in spleen cells from immunized mice (Fig. 8).

We observed a tendency in which a high percentage of CD4+ T cells from the vaccinated group produce these cytokines (IFN-γ^+^TNF-α^+^ and TNF-α^+^IL-2^+^), in comparison to the control groups, mainly to the MVA (wt) control group. Nevertheless, no statistically significant differences were observed between the groups (Kruskal–Wallis: IFN-γ^+^TNF-α^+^—*p* = 0.2225; TNF-α^+^IL-2^+^—*p* = 0.2923).

**Fig. 9:**
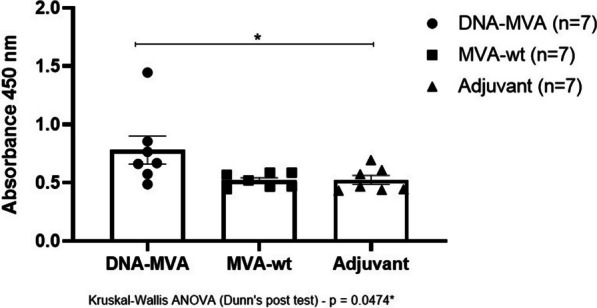
Evaluation of humoral immune response in mice immunized with the recombinant DNA-MVA through ELISA assays. Legend (symbols): Circle: Sera from mice immunized with the recombinant DNA-MVA regimen; Square: Sera from mice inoculated only with the MVA (wild type) vector; Triangle: Sera from mice inoculated only with the Addavax adjuvant. N = 7. Data represented graphically as mean and standard error of the media (SEM)

The recruitment of CD4^+^ T cells also induces the activation of antibody production. In this study, post-vaccination antibody production in BALB/c mice was evaluated through an in-house ELISA assay using the recombinant HBZ protein produced in prokaryotic system as a solid phase. IgG anti-HBZ antibody was significantly different between the vaccinated group and the adjuvant control group (Kruskal–Wallis, Dunn's post-test, *p* = 0.0474). The vaccinated group also showed a tendency to produce high level of antibodies compared to the MVA (wt) control group (Fig. 9), but despite this no statistically significant difference was observed (Kruskal–Wallis, Dunn's post-test, *p* = 0.0750).

## Discussion

The development of vaccines for diseases related to high rates of comorbidities that impact people and the health system is crucial. Due to the outcome of HTLV-1-associated diseases and the lack of effective therapies for both HAM/TSP and ATL, besides the chronic inflammation and accelerated aging in HTLV-1 infected individuals, new therapy strategies are mandatory [32]. In the HTLV context, the development of vaccines can contribute to improve the quality of life and survival of infected patients and the unburdening of health systems. This work presents a therapeutic HTLV-1 vaccine candidate based on plasmid DNA and MVA recombinant virus, both expressing an HBZ-multiepitope protein used in prime-boost heterologous protocols aimed to maximize the best delivery features presented by each vector individually [33]. Both vectors are considered stable, safe, and easy to produce. Besides to improve the immunostimulatory capacity of DNA plasmid used as prime, the MVA vector presents a precise expression control of the target gene and, as booster, promotes strong induction of cellular and humoral responses, in addition MVA vectored vaccines is proven to be safe in humans [34–38].

A therapeutic vaccine for HTLV-1 should be based on the control of the proviral load by Th-1 response [13, 39], since a high proviral load is associated with illnesses in infected individuals [40]. As proposed by MacNamara and colleagues [23] a lower proviral load is observed in individuals expressing MHC-I alleles that bind strongly to HBZ antigens, inducing a robust T CD8^+^ response that would regulate the proliferation of infected cells, consequently leading to asymptomatic conditions. Also, effective immunogens against viral infections should trigger cellular and humoral responses, while T CD4^+^ cells stimulate antibody production and sustain the effector functions of CTLs, which induces clearance of infected cells through inflammatory mediators [13, 41]. In the present study, it was possible to observe the activation of both T CD4^+^ and T CD8^+^ lymphocytes in immunized mice after in vitro stimulation with HBZ peptides, similar to the findings of Sugata et al. [17]. These stimulated T cells should have the ability to produce pro-inflammatory cytokines, such as IFN-γ and TNF-α, that act directly on infected cells and indirectly on cell differentiation and activation of other immune cells [42]. The cytokine IL-2, in addition to promoting the proliferation and differentiation of T lymphocytes primed by antigens is related to the activation of CTLs, secretion of immunoglobulins and acts as a survival factor through the induction of anti-apoptotic proteins [43]. This cytokine is characteristic of effector memory cells in order to self-stimulation, memory induction and cytotoxicity mechanisms [44].

In this study, it was evidenced the induction of polyfunctional double positive responses for TNF-α/IFN-γ and TNF-α/IL-2 both in TCD4^+^ lymphocytes and in CTLs, with TNF-α induction being prominent in the inflammatory panel. Using overlapping peptides contained in the HBZ_1–110_ region to stimulate PBMCs of Rhesus macaques Sugata et al. also obtained a response directed to the production of TNF-α and IFN-γ [17]. Moreover, the production of IL-2, a major inducer and signaling of effector T cell responses [45], was evidenced by both TCD4^+^ (Fig. 8B2) and TCD8^+^ (Fig. 5B2) cells. T CD4^+^ cells are important to have a role as auxiliary paracrine mechanism in the primary and secondary responses of CTLs, whereas T CD8+ cells are important for a robust recall response [46, 47]. Regarding to TCD8^+^ lymphocytes, it was observed a tendency in the activation of effector memory cells (CD44^+High^/CD62L^−Low^) producers of granzyme B in response to the stimulus (Fig. 7B), an important finding regarding vaccines [48]. Effector memory T cells are capable of an immediate cytotoxic response after activated by microbial antigens (through perforins and granzymes) and are therefore great mediators in the acquired immune response [13, 42]. The frequency of these cells reflects the magnitude of the initial clonal expansion induced by the vaccine formulation, which is favored by antigenic persistence during the priming phase. The prime-boost heterologous protocol used in the present work favors antigenic persistence since its “bypasses” the anti-vector response induced in the initial dose, maintaining adequate levels of stimulation throughout the boosters [42].

Cytotoxic response can act through ligands or granule-dependent mechanisms (degranulation) to eliminate the target cell [49]. Granzymes (proteases), together with perforins (pore-forming proteins), consist in the primary pathway used by CTLs to eliminate infected cells. These molecules are stored inside the lytic granules of cytotoxic cells which are secreted upon stimulation. Acting in synergy, perforins produce pores in the target cells membrane allowing the entry of granzymes, and both induce cell death through cytotoxicity mechanisms [50–52]. One of the markers of this degranulation process consists in the molecules CD107a (LAMP-1), glycoproteins associated with the membrane of lysosomal granules that are exposed on the cell surface after activation of the CTL [52]. The results raised here showed the activation of CTLs and cytotoxic response in short cultures of splenocytes from immunized mice, upon stimulation with HBZ peptides, observed by the production of granzyme B in effector memory T cells and by the expression of CD107a in T CD8^+^ cells. The overall data is consistent with a directive and effector recall response, with potential to operate actively in the elimination of infected cells and, consequently, in the reduction of the HTLV-1 proviral load.

The literature has been shown that there is no correlation between the levels of anti-HBZ antibodies and HTLV-1 proviral load, probably due to the intrinsic expression features and intracellular location of this protein during HTLV-1 infection, which naturally limit your exposure to the immune system [53, 54]. However, the ability of anti-HBZ antibodies to inhibit the spontaneous proliferation of T lymphocytes in HAM/TSP patients with no previous response to HBZ was shown, indicating a possible supporting mechanism in HTLV-1 therapy [53]. The possible activity of anti-HBZ humoral immunity might be expand to the intracellular level, since mechanisms of cytosolic neutralization by antibodies have already been demonstrated in the literature. Blocking the pathogen and their mediators, with extracellular or intracellular mechanisms, offers a protective effect by containing the spread of infection, which would indirectly reduce the proviral load [55].

The nature of the platforms used here favors humoral responses by combining the natural adjuvant effect of MVA with the synthetic compound Addavax, helping in delivery mechanisms and allowing greater antigenic persistence [42]. Mice vaccinated with the proposed plasmid and MVA vectored HBZ multiepitope vaccine candidates showed a production of IgG anti-HBZ. However, the statistical difference was observed to adjuvant control group and not to MVA control group. The reason should be, as already reported also using poxviral vectored vaccine, the low immunogenicity of HBZ antigen in mice [17].

High throughput studies have been conducted reporting that HTLV-1 infected individuals who develop ATL manifest elevated mutation burden and high degrees of mutation-based intratumor heterogeneity. Kataoka et al. [56] conducted an in-depth study characterizing an extensive spectrum of genes and pathways affected in ATL, using genome, exome, and transcriptome sequencing from a great number of HTLV-1 infected patients with ATL. Authors showed that HTLV-1 integration in ATL cells was associated with aberrant transcription, and that HBZ are generally detectable in all ATL cases, but Tax expression is rare [56]. Following, this same research group addressed clonal heterogeneity in ATL based on mutation profiles of cross-sectional whole-exome sequencing (WES) samples from 71 samples of different subtypes of ATL (smoldering, chronic, acute, and lymphoma). Results reinforced the biological and clinical significance of ITH in ATL, showing many mutations in subclones and the presence of many small clones, that are indicative of late evolution during tumor progression [57]. Thus, the high probability of the presence of resistant clones among a complex population of subclones is a relevant point in the context of new therapeutic approaches evaluation. As ATL cells can be considered as HTLV-1 infected cells which obtained loads of mutations and transformed in vivo, and due its capacity of escape to the host immune surveillance, and losing the dependency for survival and proliferation, the development of therapeutics approaches should consider the capacity of these tools target both infected and transformed cells. At this point and considering the stage of this preclinical assay presented here, this answer cannot be achieved. However, it is important to highlight that one of the peptides included in the HBZ multiepitope vectors (Fig. 1—HBZ_157–176_) used in the prime-boost protocol was described by Sugata and colleagues [17] using a recombinant *Vaccinia virus* vaccine expressing HBZ. They proposed HBZ_157-176_ as a candidate peptide for future vaccine developments, after observe that dendritic cells pulsed with this peptide could generate HBZ-specific CTLs from human CD8^+^ T cells and that HBZ-specific effector cells improved the survival rate of an ATL mouse model [17], suggesting that a therapeutic vaccine targeting HBZ might be useful as a tool to control or prevent ATL development in HTLV-1 infected individuals.

Related to therapeutic use in future of the vaccine proposed in this preclinical test, in HAM/TSP HBZ-specific immune response is negatively correlated with disease progression. It was reported that the presence of an HBZ-specific immune response was associated with reduced CD4+ T cell activation in HAM/TSP patients, and decreased lymphoproliferation in the ex-vivo PBMCs of HAM/TSP patients [53].

One of the limitations of the present study is the use of peptides predicted in silico for human MHC-I alleles in a BALB/c model, which may have generated suboptimal responses. Furthermore, other complementary approaches aiming at evaluating the immune response and associated mechanisms will be included in future studies. Nonetheless, given the results described above, the general immune context observed in the present study is consistent with the findings reported in the literature [17], likewise corroborating that booster doses of HBZ antigens can generate robust T cell responses.

## Conclusions

The immunogenic response obtained in this preclinical assay using prime-boost heterologous protocol with HBZ multiepitope protein vectored in DNA plasmid and recombinant MVA showed the induction of polyfunctional double positive responses for IFN-γ and TNF-α, and for TNF-α and IL-2 both in T CD4^+^ and T CD8^+^ lymphocytes and the activation of CTLs and the cytotoxic response. The overall data are consistent with a directive and effector recall response, with potential to operate actively in the elimination of infected cells and, consequently, to reduce proviral load and highlights the potential of these constructions as HTLV-1 vaccine candidates. New tests in humanized mice and primates using the proposed vaccine protocol are in progress in our research group and will contribute for the next steps in the search for a vaccine against HTLV-1 based on HBZ.

### Supplementary Information

**Additional file 1: Figure S1.** Design of the recombinant pcDNA3.1(+)-HBZ. Illustrative figure of the pcDNA3.1(+)-HBZ plasmid used in the immunization assays presenting the HBZ multiepitope sequence. **Figure S2.** Selection of transforming colonies of pcDNA3.1(+)-HBZ plasmid by PCR and enzymatic restriction. **A** PCR (1) Ladder 1 Kb (Invitrogen); (2–5) Mini-preparations of pcDNA3.1(+)-HBZ. Expected fragment (2702 bp) that encodes the HBZ construction; **B** Enzyme restriction-1: Ladder 1 Kb Invitrogen; (3 and 4) Control plasmid not digested; (6 and 8) Plasmid samples double digested with *NheI* and *SmaI*. Expected fragment (1437 bp) that encodes the HBX-multiepitope protein. **Figure S3.** Endotoxin purified pcDNA3.1(+)-HBZ. PCR product. (1) Ladder 1 Kb (Bioron); (2–4) PCR product of pcDNA3.1(+)-HBZ multiepitope endotoxin purified. Expected fragment (2702 bp) that encodes the HBZ construction. (5) Positive control—Mini-prep of pcDNA3.1(+)-HBZ. (6) Negative control.

## Data Availability

The datasets supporting the conclusions of this article are included within the article and its additional files.

## Abbreviations

ATL: Adult T-cell leukemia*/*lymphoma
GFP: Green fluorescent protein
HAM/TSP: HTLV-I-associated myelopathy/tropical spastic paraparesis
HBZ: HTLV-1 basic leucine zipper (bZIP) factor
HTLV-1: Human T-lymphotropic virus 1
ICS: Intracellular cytokine staining
MVA: Modified *Vaccinia virus* Ankara
MVA-HBZ: MVA expressing a multiepitope protein based on HBZ
MOI: Multiplicity of infection
PFU: Plaque-forming unit
VACV: *Vaccinia virus*

## References

[1] King-Robson J, Hampton T, Rosadas C (2022). HTLV-1 encephalitis. Pract Neurol.

[2] Proietti ABFC (2015). Cadernos hemominas—HTLV.

[3] Cook LB, Elemans M, Rowan AG (2013). HTLV-1: persistence and pathogenesis. Virology.

[4] Firouzi S, López Y, Suzuki Y (2014). Development and validation of a new high-throughput method to investigate the clonality of HTLV-1-infected cells based on provirus integration sites. Genome Med.

[5] Firouzi S, Farmanbar A, Nakai K (2017). Clonality of HTLV-1-infected T cells as a risk indicator for development and progression of adult T-cell leukemia. Blood Adv.

[6] Farmanbar A, Kneller R, Firouzi S (2019). RNA sequencing identifies clonal structure of T-cell repertoires in patients with adult T-cell leukemia/lymphoma. NPJ Genom Med.

[7] Schierhout G, Mcgregor S, Gessain A (2020). Association between HTLV-1 infection and adverse health outcomes: a systematic review and meta-analysis of epidemiological studies. Lancet Infect Dis.

[8] Ramos JM, De Mendoza C, Soriano V (2020). Spanish HTLV network. HTLV-1 infection and health outcomes. Lancet Infect Dis.

[9] Santana CS, Andrade FO, Da Silva GCS (2023). Advances in preventive vaccine development against HTLV-1 infection: a systematic review of the last 35 years. Front Immunol.

[10] Kabiri M, Sankian M, Sadri K, Tafaghodi M (2018). Robust mucosal and systemic responses against HTLV-1 by delivery of multi-epitope vaccine in PLGA nanoparticles. Eur J Pharm Biopharm.

[11] Ishii H, Nakamura-Hoshi M, Shu T (2022). Sendai virus particles carrying target virus glycoproteins for antibody induction. Vaccine.

[12] Kabiri M, Sankian M, Hosseinpour M (2018). The novel immunogenic chimeric peptide vaccine to elicit potent cellular and mucosal immune responses against HTLV-1. Int J Pharm.

[13] Seighali N, Shafiee A, Rafiee MA (2023). Human T-cell lymphotropic virus type 1 (HTLV-1) proposed vaccines: a systematic review of preclinical and clinical studies. BMC Infect Dis.

[14] Lucchese G, Jahantigh HR, De Benedictis L (2021). An epitope platform for safe and effective HTLV-1-immunization: potential applications for mRNA and peptide-based vaccines. Viruses.

[15] García-Arriaza J, Esteban M (2014). Enhancing poxvirus vectors vaccine immunogenicity. Hum Vaccin Immunother.

[16] Franchini G, Tartaglia J, Markham P (1995). Highly attenuated HTLV type env poxvirus vaccines induce protection against a cell-associated HTLV type I challenge in rabbits. AIDS Res Hum Retrovir.

[17] Sugata K, Yasunaga J, Mitobe Y (2015). Protective effect of cytotoxic T lymphocytes targeting HTLV-1 bZIP factor. Blood.

[18] Perdiguero B, Pérez P, Marcos-Villar L (2023). Highly attenuated poxvirus-based vaccines against emerging viral diseases. J Mol Biol.

[19] Ohshima K (2015). Molecular pathology of adult T-cell leukemia/lymphoma. Oncology.

[20] Tanaka-Nakanishi A, Yasunaga J, Takai K (2014). HTLV-1 bZIP factor suppresses apoptosis by attenuating the function of FoxO3a and altering its localization. Cancer Res.

[21] Saito M, Matsuzaki T, Satou Y (2009). In vivo expression of the HBZ gene of HTLV-1 correlates with proviral load, inflammatory markers and disease severity in HTLV-1 associated myelopathy/tropical spastic paraparesis (HAM/TSP). Retrovirology.

[22] Cavanagh MH, Landry S, Audet B (2006). HTLV-I antisense transcripts initiating in the 3'LTR are alternatively spliced and polyadenylated. Retrovirology.

[23] Macnamara A, Rowan A, Hilburn S (2010). HLA class I binding of HBZ determines outcome in HTLV-1 infection. PLoS Pathog.

[24] Suemori K, Fujiwara H, Ochi T (2009). HBZ is an immunogenic protein, but not a target antigen for human T-cell leukemia virus type 1-specific cytotoxic T lymphocytes. J Gen Virol.

[25] Wen YM, Wang YX (2009). Biological features of hepatitis B virus isolates from patients based on full-length genomic analysis. Rev Med Virol.

[26] Dipti CA, Jain SK, Navin K (2006). A novel recombinant multiepitope protein as a hepatitis C diagnostic intermediate of high sensitivity and specificity. Protein Expr Purif.

[27] Bisht H, Roberts A, Vogel L (2004). Severe acute respiratory syndrome coronavirus spike protein expressed by attenuated Vaccinia virus protectively immunizes mice. Proc Natl Acad Sci USA.

[28] Daian e Silva DSO, Pinho TMG, Rachid MA (2019). The perennial use of the green fluorescent protein marker in a live vaccinia virus Ankara recombinant platform shows no acute adverse effects in mice. Braz J Microbiol.

[29] Daian e Silva DSO, Barbosa-Stancioli EF, Coelho-Dos-Reis JGA (2020). Short communication: a modified *Vaccinia*
*virus* Ankara-based *Porcine*
*circovirus* 2 vaccine elicits strong antibody response upon prime-boost homologous immunization in a preclinical model. Braz J Microbiol.

[30] Flesch IE, Wong YC, Tscharke DC, Isaacs SD (2012). Analyzing CD8 T cells in mouse models of poxvirus infection. Vaccinia virus and poxvirology: methods and protocols (methods in molecular biology).

[31] Quinan BR, Flesch IEA, Pinho TMG (2014). An intact signal peptide on dengue virus E protein enhances immunogenicity for CD8+ T cells and antibody when expressed from modified *Vaccinia* Ankara. Vaccine.

[32] Willems L, Hasegawa H, Accolla R (2017). Reducing the global burden of HTLV-1 infection: an agenda for research and action. Antiviral Res.

[33] Nascimento IP, Leite LC (2012). Recombinant vaccines and the development of new vaccine strategies. Braz J Med Biol Res.

[34] Kutzler MA, Weiner DB (2008). DNA vaccines: Ready for prime time?. Nat Rev Genet.

[35] Beláková J, Horynová M, Krupka M (2007). DNA vaccines: Are they still just a powerful tool for the future?. Arch Immunol Ther Exp (Warsz).

[36] Quinan BR, Daian DSO, Coelho FM (2014). Modified vaccinia virus Ankara as vaccine vectors in human and veterinary medicine. Future Virol.

[37] Drexler I, Staib C, Sutter G (2004). Modified *Vaccinia* virus Ankara as antigen delivery system: How can we best use its potential?. Curr Opin Biotechnol.

[38] Natalini A, Simonetti S, Sher C (2022). Durable CD8 T cell memory against SARS-CoV-2 by prime/boost and multi-dose vaccination: considerations on inter-dose time intervals. Int J Mol Sci.

[39] Ratner L (2022). A role for an HTLV-1 vaccine?. Front Immunol.

[40] Vine AM, Heaps AG, Kaftantzi L (2004). The role of CTLs in persistent viral infection: cytolytic gene expression in CD8^+^ lymphocytes distinguishes between individuals with a high or low proviral load of human T cell lymphotropic virus type 1. J Immunol.

[41] Jahantigh HR, Stufano A, Koohpeyma F (2023). Recombinant GPEHT fusion protein derived from HTLV-1 proteins with alum adjuvant induces a high immune response in mice. Vaccines (Basel).

[42] Siegrist CL, Orenstein WA, Offit PA, Edwards KM, Plotkin SA (2018). Vaccine immunology. The Netherlands: Plotkin's vaccines.

[43] Asao H (2014). Interleukin-2. Japan: reference module in biomedical sciences.

[44] Bendickova K, Fric J (2020). Roles of IL-2 in bridging adaptive and innate immunity, and as a tool for cellular immunotherapy. J Leukoc Biol.

[45] Kalia V, Sarkar S (2018). Regulation of effector and memory CD8 T cell differentiation by IL-2-A balancing act. Front Immunol.

[46] Toumi R, Yuzefpolskiy Y, Vegaraju A (2022). Autocrine and paracrine IL-2 signals collaborate to regulate distinct phases of CD8 T cell memory. Cell Rep.

[47] Feau S, Arens R, Togher S (2011). Autocrine IL-2 is required for secondary population expansion of CD8(+) memory T cells. Nat Immunol.

[48] Kaech SM, Wherry EJ, Ahmed R (2002). Effector and memory T-cell differentiation: implications for vaccine development. Nat Rev Immunol.

[49] Betts MR, Brenchley JM, Price DA (2003). Sensitive and viable identification of antigen-specific CD8+ T cells by a flow cytometric assay for degranulation. J Immunol Methods.

[50] Boivin W, Cooper D, Hiebert P (2009). Intracellular *versus* extracellular granzyme B in immunity and disease: challenging the dogma. Lab Investig.

[51] Hay ZLZ, Slansky JE (2022). Granzymes: the molecular executors of immune-mediated cytotoxicity. Int J Mol Sci.

[52] Schulte I, Zhang EJ, Meng ZJ (2008). Recent advances in research on hepadnaviral infection in the woodchuck model. Virol Sin.

[53] Enose-Akahata Y, Abrams A, Massoud R (2013). Humoral immune response to HTLV-1 basic leucine zipper factor (HBZ) in HTLV-1-infected individuals. Retrovirology.

[54] Shiohama Y, Naito T, Matsuzaki T (2016). Absolute quantification of HTLV-1 basic leucine zipper factor (HBZ) protein and its plasma antibody in HTLV-1 infected individuals with different clinical status. Retrovirology.

[55] Mallery DL, Mcewan WA, Bidgood SR (2010). Antibodies mediate intracellular immunity through tripartite motif-containing 21 (TRIM21). Proc Natl Acad Sci USA.

[56] Kataoka K, Nagata Y, Kitanaka A (2015). Integrated molecular analysis of adult T cell leukemia/lymphoma. Nat Genet.

[57] Farmanbar A, Firouzi S, Makalowski W, Kneller R, Iwanaga M, Utsunomiya A, Nakai K, Watanabe T (2018). Mutational intratumor heterogeneity is a complex and early event in the development of adult T-cell leukemia/lymphoma. Neoplasia.

