# Supplementary material for: Preclinical assessment of an anti-HTLV-1 heterologous DNA/MVA vaccine protocol expressing a multiepitope HBZ protein
Source: Virol J. 2023 Dec 19;20:304. doi: 10.1186/s12985-023-02264-z (PMC10731796; doi:10.1186/s12985-023-02264-z)
Supplement: Supplementary file 1 — Additional file 1: Figure S1. Design of the recombinant pcDNA3.1(+)-HBZ. Illustrative figure of the pcDNA3.1(+)-HBZ plasmid used in the immunization assays presenting the HBZ multiepitope sequence. Figure S2. Selection of transforming colonies of pcDNA3.1(+)-HBZ plasmid by PCR and enzymatic restriction. A PCR (1) Ladder 1 Kb (Invitrogen); (2–5) Mini-preparations of pcDNA3.1(+)-HBZ. Expected fragment (2702 bp) that encodes the HBZ construction; B Enzyme restriction-1: Ladder 1 Kb Invitrogen; (3 and 4) Control plasmid not digested; (6 and 8) Plasmid samples double digested with NheI and SmaI. Expected fragment (1437 bp) that encodes the HBX-multiepitope protein. Figure S3. Endotoxin purified pcDNA3.1(+)-HBZ. PCR product. (1) Ladder 1 Kb (Bioron); (2–4) PCR product of pcDNA3.1(+)-HBZ multiepitope endotoxin purified. Expected fragment (2702 bp) that encodes the HBZ construction. (5) Positive control—Mini-prep of pcDNA3.1(+)-HBZ. (6) Negative control. [file 12985_2023_2264_MOESM1_ESM.docx]

**SUPPLEMENTARY FIGURES**

**FIGURE S1: Design of the recombinant pcDNA3.1(+)-HBZ.** Illustrative figure of the pcDNA3.1(+)-HBZ plasmid used in the immunization assays presenting the HBZ multiepitope sequence.


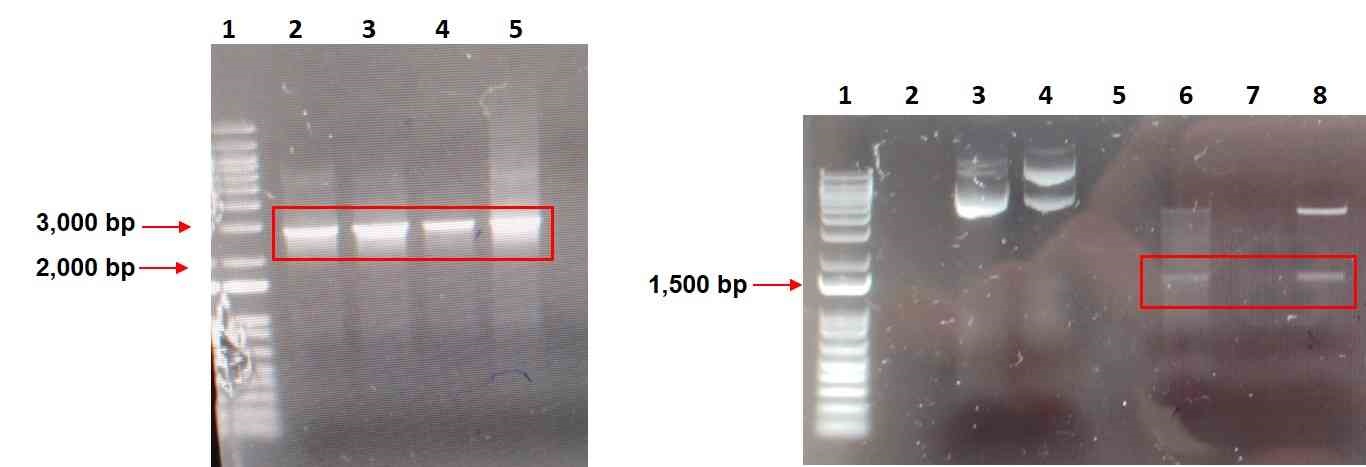


**A**

**B**

**FIGURE S2: Selection of transforming colonies of pcDNA3.1(+)-HBZ plasmid by PCR and enzymatic restriction. A: PCR** – **1:** Ladder 1 Kb (Invitrogen); **2 to 5:** Mini-preparations of pcDNA3.1(+)-HBZ. Expected fragment (2,702 bp) that encodes the HBZ construction; **B: Enzyme restriction** - **1:** Ladder 1 Kb Invitrogen; **3 and 4:** Control plasmid not digested; **6 and 8:** Plasmid samples double digested with *NheI* and *SmaI*. Expected fragment (1,437 bp) that encodes the HBX-multiepitope protein.


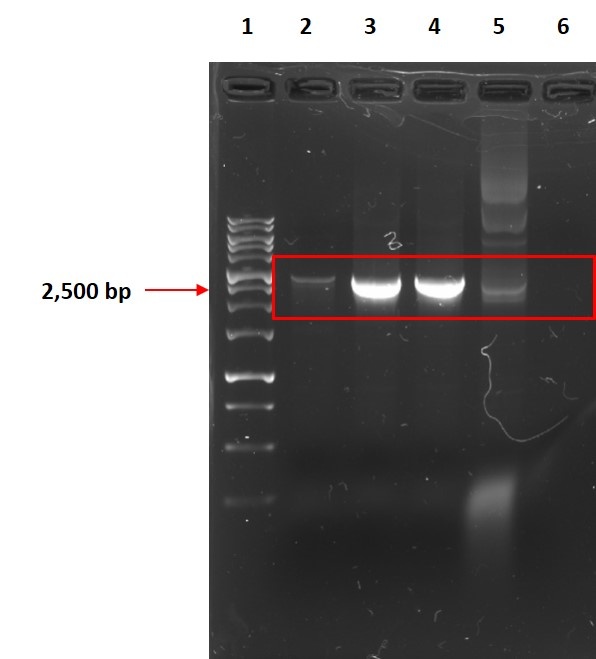


**FIGURE S3: FIGURE S3: Endotoxin purified pcDNA3.1(+)-HBZ.** PCR product. **1:** Ladder 1 Kb (Bioron); **2 to 4:** PCR product of pcDNA3.1(+)-HBZ multiepitope endotoxin purified. Expected fragment (2,702 bp) that encodes the HBZ construction. **5:** Positive control - Mini-prep of pcDNA3.1(+)-HBZ. **6:** Negative control.
